# Vacuoles in Bryophytes: Properties, Biogenesis, and Evolution

**DOI:** 10.3389/fpls.2022.863389

**Published:** 2022-06-07

**Authors:** Hao-ran Liu, Chao Shen, Danial Hassani, Wan-qi Fang, Zhi-yi Wang, Yi Lu, Rui-liang Zhu, Qiong Zhao

**Affiliations:** ^1^School of Life Sciences, East China Normal University, Shanghai, China; ^2^Institute of Eco-Chongming, Shanghai, China

**Keywords:** bryophyte, endomembrane system, vacuole, biogenesis, regulator, evolution

## Abstract

Vacuoles are the most conspicuous organelles in plants for their indispensable functions in cell expansion, solute storage, water balance, etc. Extensive studies on angiosperms have revealed that a set of conserved core molecular machineries orchestrate the formation of vacuoles from multiple pathways. Usually, vacuoles in seed plants are classified into protein storage vacuoles and lytic vacuoles for their distinctive morphology and physiology function. Bryophytes represent early diverged non-vascular land plants, and are of great value for a better understanding of plant science. However, knowledge about vacuole morphology and biogenesis is far less characterized in bryophytes. In this review, first we summarize known knowledge about the morphological and metabolic constitution properties of bryophytes' vacuoles. Then based on known genome information of representative bryophytes, we compared the conserved molecular machinery for vacuole biogenesis among different species including yeast, mammals, *Arabidopsis* and bryophytes and listed out significant changes in terms of the presence/absence of key machinery genes which participate in vacuole biogenesis. Finally, we propose the possible conserved and diverged mechanism for the biogenesis of vacuoles in bryophytes compared with seed plants.

## Introduction

The endomembrane system (ES), which is composed of organelles and their connections termed as membrane trafficking, is found only in eukaryotes. This remarks its evolutionary and functional importance to eukaryotic life. Based on the type of eukaryotic cells, the endomembrane trafficking pathway, engage various organelles including, endoplasmic reticulum (ER), Golgi, *trans*-Golgi network/early endosome (TGN/EE), multivesicular body/late endosome (MVB/LE), and lysosome/vacuole (Cui et al., 2020; Hu et al., 2020). Extant eukaryotes, as descendants from the last eukaryotic common ancestor (LECA), although exhibit enormous distinctions, share several sets of conserved-core molecular machineries (CCMMs) which orchestrate the basic ES-related cellular events. Here in this review, we mainly focus on the CCMMs orchestrating vacuole biogenesis across kingdoms.

Different from animal cells, plant cells have evolved several fundamental unique cellular structures, such as cell wall, chloroplast, central vacuole, and plasmodesmata to cope with changing signals from internal and external stimuli. The membrane trafficking pathway in plants is mainly divided into the biosynthetic secretory pathway, the endocytic pathway, and the vacuole trafficking pathway (Aniento et al., 2022). In plant cells, the vacuole is an important part of the vacuole trafficking pathway. Except for protein storage and degradation, vacuoles store water, accommodate many other metabolites, and maintain the basic cell physiological activities such as pH and turgor pressure (Wolf et al., 2010; Shimada et al., 2018; Tan et al., 2019). In addition, vacuoles play significant roles in plant protection against biotic and abiotic stress. Tracheophytes and bryophytes share a common ancestor about 500 million years ago and constitute the two deeply diverged land plant groups. Therefore, it is of great importance to characterize plant-unique membrane-related regulatory paradigm by referring to bryophyte plant species.

Bryophytes are a group of terrestrial, non-vascular plant species, which represent early-diverging embryophytes. In bryophytes, for instance, storage of heavy metals such as Fe, Al, and Cd in vacuoles is a protective strategy to reduce their adverse effect (Thiébaut et al., 2008; Zhang et al., 2018; Tan et al., 2019). Similarly, under intense UV-B conditions, vacuoles accumulate UV-absorbing compounds, mostly phenolic, in both angiosperms and bryophytes (Wolf et al., 2010; Fabón et al., 2012). These indeed highlight the importance of vacuoles and their conserved evolutionary biogenesis among various plant species.

Here, by referring to vacuole-related research on bryophytes, including both experimental studies and our own phylogenetic studies with comparative genomic approaches, we first highlighted the most current morphological and metabolic constitution properties of bryophytes' vacuoles; and then we compared the conserved molecular machinery for vacuole biogenesis among different bryophytes species with *Arabidopsis*, and listed out important presence/absence events of key proteins which participate in vacuole biogenesis; finally we propose possible interpretations of conserved and diverse vacuole biogenesis and function in bryophytes compared with seed plants.

## Bryophytes' Morphology and Life Cycle

**Figure 1:**
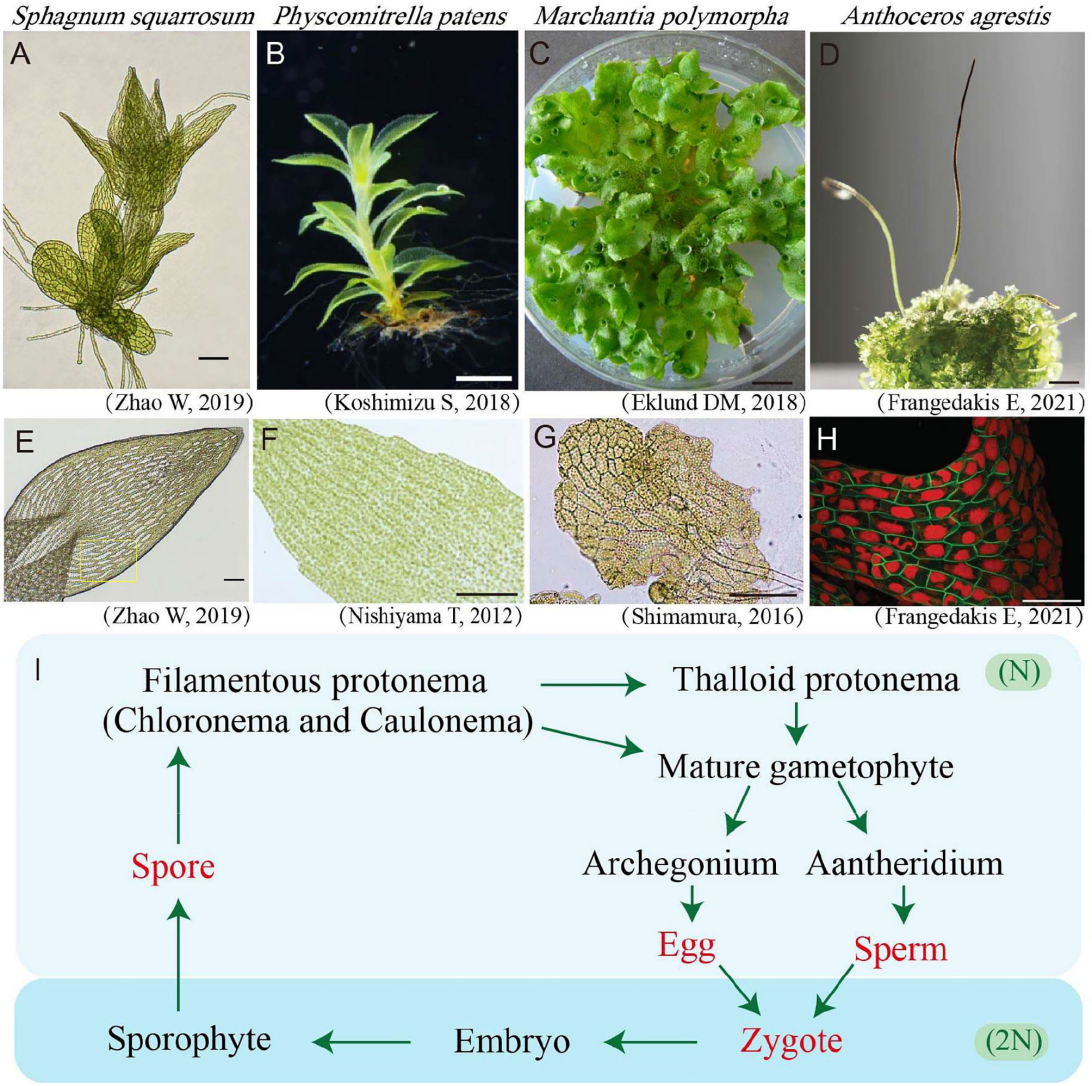
Gametophyte and leaves/thallus cells of bryophytes. **(A)** Light microscopy photos of young *Sphagnum squarrosum* gametophyte. Bar = 100 μm. **(B)** Photo of *Physcomitrella patens's* mature gametophyte (Koshimizu et al., 2018). Bar = 1 mm. **(C)** Photo of *Marchantia polymorpha's* mature gametophyte (Eklund et al., 2018). Bar = 1 mm. **(D)** Photo of *Anthoceros agrestis* gametophyte (the bottom area) and sporophytes (the top slender area). Bar = 3 mm. **(E)** Photo of a leaf with green and white cells in *S. squarrosum* (Li et al., 2019). Bar = 100 μm. **(F)** Light microscopy photo of a leaf in *P. patens* (Nishiyama et al., 2012). Bar = 100 μm. **(G)** Light microscopy photo of young thallus cells of *M. polymorpha* (Shimamura, 2016). Bar = 100 μm. **(H)** Confocal fluorescence microscopy image of *A. agrestis* gametophyte cells in which the plasma membrane is shown in green and the plastid is shown in red (Frangedakis et al., 2021). Bar = 100 μm. **(I)** Life cycle of bryophytes. The words in red denote single cell stages while the words in black denote multicellular stages. The light blue background indicates the haploid (N) stages, while the dark blue background indicates the diploid (2N) stages of bryophytes.

Bryophytes are divided into three types, namely, *Hepaticopsida* (Liverworts), *Bryopsida* (Mosses), and *Anthocerotopsida* (Hornworts) (Klips, 2016). They lack flowers, seeds, and true root systems, which are quite different from the model seed plant *Arabidopsis thaliana* (Figures 1A–H).

The life cycle of bryophytes consists of two alternating generations, namely, the haploid gametophyte and the diploid sporophyte, of which the gametophyte is the dominant generation (Figure 1I). The haploid gametophyte stage begins from a single cell by spore (single cell) germination, which will be further developed into a 2-D multiple-cell stage termed as protonemata. In mosses, the protonemata are divided into the early stage chloronema and late stage caulonema which possess higher and lower chloroplast density, respectively (Cove et al., 1997). Further in most bryophytes, the 2-D multicellular stage protonema will develop more complicated 3-D multicellular organs including thalloid, rhizoids, and aerial filaments, finally leading to a mature leafy or thallus gametophyte individual (Figures 1A–D). Bryophytes reproduce both in sexual and asexual (vegetative) ways. For sexual reproduction, bryophytes use two types of reproductive organs, namely the antheridium for eggs, and the archegonium for sperms. The mixing of genetic material from parental plants is achieved via egg fertilization in the antheridium. The fertilized egg will further develop into the diploid sporophyte stage (Shimamura, 2016) (Figure 1I). The continued mitosis division of the embryo will give rise to a mature sporophyte containing foot, seta, and capsule, which will further produce and release haploid spores through meiosis (Frangedakis et al., 2021). For vegetative reproduction, various ways have been reported for different bryophytes species, and almost all bryophytes could reproduce vegetatively.

## Different Populations of Vacuoles in Bryophytes

It has been known that vacuoles do not have a fixed morphology and size, while its structure varies according to the growth stage or the environmental conditions (Richards et al., 2010; Cao et al., 2020; Cui et al., 2020). In seed plants, like in *A. thaliana*, vacuoles are mainly divided into lytic vacuoles (LVs) and protein storage vacuoles (PSVs) due to their distinctive morphologies and physiology functions (Paris et al., 1996; Feeney et al., 2013; Cui et al., 2020). LVs contain lytic enzymes and keep an acidic pH (Kriegel et al., 2015), while PSVs store lots of proteins in seeds and keep a neutral pH (Isayenkov, 2014; Zhang et al., 2021). LVs and PSVs are interchangeable during different developmental stages (Feeney et al., 2018).

Similar to any plants, the bryophytes cells are of different shapes and sizes with different subcellular structures during the growth, development, and in response to changing environment. Numerous researches with light and electron microscopic images of different bryophyte cells indicate the presence of multiple vacuole populations, including vacuoles of different sizes, vacuoles of different pHs, and vacuoles of different contents, but no PSV is reported.

Vacuoles of different sizes have been reported to different species including both moss and liverwort. During the mature gametophyte stage, several mosses including, *Sphagnum recurvum, Sphagnum cuspidatum*, and *Sphagnum palustre*, bear large central vacuoles in thick walled sterome cells, while the internal thin-walled cells possess abundant number of small vacuoles (Ligrone and Duckett, 1998). In contrast to the gametophyte stage, in some mosses such as *Ephemerum cohaerens* the small vacuole is dominant in basal foot cells, while in *Timmiella barbuloides* big vacuole in the tapetum cells is formed after cytokinesis in sporophyte (Gambardella et al., 1994; Yip and Rushing, 1999). In liverworts, for example, in *Asterella wilmsii*, central large vacuoles are present in the chlorenchyma cells besides air chambers and the young inner thallus cells, while the small vacuoles lay near the fully differentiated thallus apex (Ligrone and Duckett, 1994). The study in liverwort *Lophozia ventricosa* and *Bazzania trilobata* found that meristematic cells of the shoot apex lacked vacuoles, while the vacuoles in a mature leaf cell were large (Pihakaski, 1968).

Vacuoles maintain the cellular pH via the pumping of protons through the action of V-ATPase into the vacuole lumen, making it acidic (Kriegel et al., 2015). Inside vacuoles, under these acidic conditions, acid hydrolase enzymes break down large molecules (Rojo et al., 2003). In seed plants, LVs are the most acidic compartment (Shen et al., 2013). In addition to pH adjustment, the V-ATPase is crucial for a plethora of cellular functions including both endocytic and secretory pathways (Dettmer et al., 2006). In bryophytes, vacuoles of similar appearance but different pHs have been reported. For example, large central vacuoles in protonema and rhizoids cells of *P. patens* are acidic vacuoles (AV), while non-acidic vacuoles (NV) coexist in the same protonema apical cell (Ayachi, 2013). This highlights that vacuoles of different pH exist in bryophytes.

Vacuoles are not only on the structure basis of plant cells but also play crucial roles in the transportation and storage of more than 200,000 secondary metabolites, such as phenolic, terpenoids, and alkaloids compounds (Francisco and Martinoia, 2018). Most of these metabolites are considered as a defensive tool against biotic and abiotic stress which are released upon cell destruction. For instance, under UV-B radiation, bryophytes could synthesize and accumulate UV-absorbing compounds (UVAC) to protect themselves. These phenolic compounds include two types, namely, the soluble SUVAC and the cell-wall bound WUVAC. Vacuoles-localized SUVACs are more responsive to the elevation in UV-B level in comparison to WUVACs (Fabón et al., 2012; Hespanhol et al., 2014; Monforte et al., 2015; Soriano et al., 2019).

Besides UV-B absorbing compounds, there are other groups of secondary metabolites which are synthesized and accumulate in bryophytes. Flavonoids and terpenoids are among the most abundant groups of natural bioactive compounds which are not only beneficial for the plant itself but also play a health-promoting role for humans (Hassani et al., 2020). For instance, hundreds of compounds are extracted, and over 40 aromatic compounds and terpenoids have been discovered in *M. polymorpha* (Asakawa and Ludwiczuk, 2013). However, how vacuoles may contribute to these groups of secondary metabolites are largely unknown. Recent studies in *M. polymorpha* highlighted that the secretory pathway of the endomembrane system contributes to the formation of the specialized organelle called the oil body, which is responsible for terpenoid storage in liverwort. The production of secondary metabolites is a crucial evolutionary trait that the plants have adopted to enhance their survival rate against harsh environmental factors. In this regard, the co-evolution of the endomembrane system and their regulatory network with secondary metabolites production and storage is indispensable in plants.

## Vacuole Dynamics in Bryophytes

Plant vacuoles are also highly dynamic, which may undergo continuous coordinated cycles of fusion and fission actions as observed in guard cells, or perform convolution-like actions to transform a large central vacuole into interconnected bubbles or into separated fragmented small vacuoles (Cui et al., 2020). Vacuole dynamics allow vacuoles to change in size, shape, and number during cell division and in response to growth and environmental signals (Kimata et al., 2019). In Arabidopsis, studies have shown that the SNAREs VTI11 and VTI12 are major players in tonoplast remodeling and transport to the vacuole, respectively (Sanmartin et al., 2007). Meanwhile, excellent studies in Arabidopsis have recently demonstrated that vacuole-actin interaction severely impacts vacuole dynamics and vacuole morphologies (Deeks et al., 2012; Scheuring et al., 2016). Also, it has been found that Vacuole-actin adaptors Networked 4 are regulating vacuole size and morphology (Kaiser et al., 2019). The cell wall, as a rigid container outside of protoplast, was recently shown to affect vacuole size and shape through cellular signal communication between cell wall and vacuole (Dunser et al., 2019). In addition, plant hormone auxin has been shown to change vacuole morphology through the regulation of SNARE protein abundance (Lofke et al., 2015). Studies in BY2 cells have also revealed the dynamic change of vacuole size and morphology during different stages of the cell cycle (Kutsuna et al., 2003). Tonoplast localized water channel protein TIPs are traditionally used as markers to distinguish different populations of vacuoles in a variety of seed plants (Gattolin et al., 2009, 2010), but not in bryophytes.

Similar vacuole dynamics have been observed in bryophytes. In moss, for instance, in *P. patens* a large central vacuole occupies the cellular space in apical and subapical cells during chloronema and caulonema stages, respectively, while the large vacuole will transform into several small vacuoles in apical cells during caulonema stage (Jensen and Jensen, 1984; Pressel et al., 2008; Ayachi, 2013; Radin et al., 2021). Pressel et al. reported the presence of a large central vacuole in either undifferentiated or early-stage differentiated caulonema of several mosses, including *Funaria hygrometrica, Tortula muralis*, and *Bartramia pomiformi*. However, the presence of cytoplasmic strands in these mosses leads to the separation of large vacuoles into several small vacuoles during late-stage differentiation of caulonema and rhizoid (Pressel et al., 2008). In addition, liverwort *Polytrichum juniperinum* and *Hypnum jutlandicum* possess several transparent vacuoles in younger spermatids and a large vacuole opening (VO) onto the spermatid surface during the spermatogenesis stage (Miller and Duckett, 1986).

In addition to the growth stage, environmental stimuli will greatly influence the shape and structure of vacuoles in bryophytes. For instance, the abscisic acid (ABA) treatment disintegrated large vacuoles into smaller ones in protonema of moss including *P. patens* and *F. hygrometrica* (Schnepf and Reinhard, 1997; Nagao et al., 2005; Arif et al., 2019). In the liverwort *Southbya nigrella*, the large central vacuoles were broken into small vacuoles in dehydrated leaf and cortical stem cells (Pressel et al., 2009). Another research also observed the small vacuoles in moss *D. plumbicola* dehydrated protonema cells and the frozen cells also contain many small vacuoles (Rowntree et al., 2007). Similarly, vacuoles in *M. polymorpha*, cells fragmented into small vacuoles when treated with ABA, and similar phenomena appeared when treated with sucrose (Akter et al., 2014). A similar ABA effect on vacuoles has been observed in moss *Ditrichum plumbicola*, and vacuoles in protonema cells fragmented into small vacuoles under ABA and sucrose treatment (Rowntree et al., 2007). It is worth noting that, however, contradicted effects have been reported in another recent independent study. In M. *polymorpha* cells, the vacuole volume increased with 1 μM ABA, with water deficit (with relative humidity maintained at 60%), or with NaCl (Godinez-Vidal et al., 2020). The reason why the vacuoles had opposite phenotypes under the similar treatment may be due to different growth and physiological status.

## Changes in The Molecular Toolkit Controlling Vacuole Biogenesis in Bryophytes

Similar to every other mechanism taking place in living organisms, the biogenesis and function of vacuoles are also controlled by the molecular machinery of the cells. In *A. thaliana*, main vacuolar biogenesis pathways are regulated by different functional protein complexes, such as the endosomal sorting complex required for transport (ESCRT) (Gao et al., 2017), adaptor protein complexes (APs) (Zwiewka et al., 2011; Wang et al., 2014), RAB5 (Sohn et al., 2003; Goh et al., 2007), RAB7 (Cui et al., 2014), Phosphoinositide 3-kinase (PI3K), homotypic fusion and protein sorting (HOPS) and soluble NSF attachment protein receptor (SNARE) (Takemoto et al., 2018). In many plants, the ESCRT complex mediates the MVB biogenesis and protein sequestering into intraluminal vesicles (ILVs) (Henne William et al., 2011; Cui et al., 2016), while APs regulate protein trafficking to both LVs and PSVs (Zhang et al., 2021). In contrast, RAB5, RAB7, and SNAREs are involved in vesicles transporting and vesicles fusion with vacuoles, respectively (Wang et al., 2017; Minamino and Ueda, 2019). Most of these regulators are conserved in plants and play key roles in angiosperms and bryophytes' endomembrane trafficking pathways (Kanazawa et al., 2016). For instance, several members of SNAREs family, such as SYP8, SEC20, USE1, and SEC22, are localized to the ER in both *A. thaliana* and *Marchantia polymorpha* (Uemura et al., 2004; Kanazawa et al., 2016). However, some members of SNAREs family including SYP5, SYP2, and VAMP71, which have one copy in *M. polymorpha* are exclusively localized to the vacuole. In turn, *A. thaliana* contains two, three, and four copies of these proteins, which are localized to Golgi or vacuoles, respectively (Kanazawa et al., 2016).

**Figure 2:**
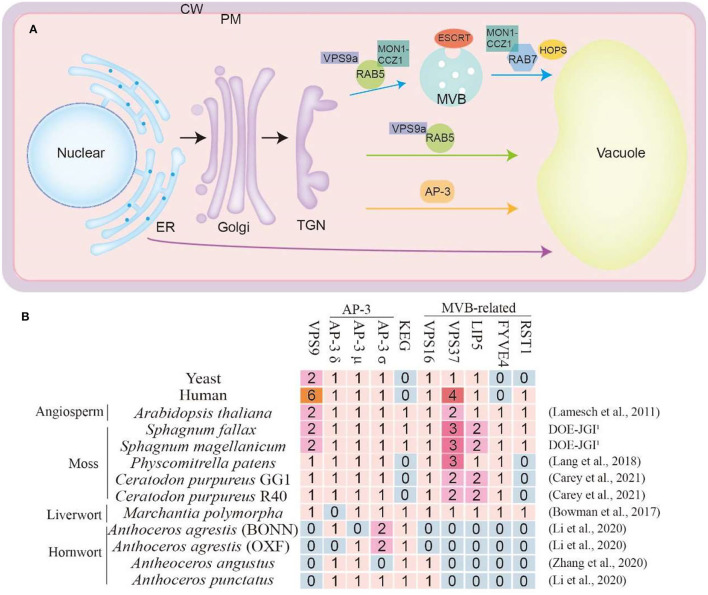
**(A)** Vacuole trafficking pathway in plants. The blue arrows indicate the pathway mediated by RAB5 and RAB7. The green arrows indicate the vacuole trafficking pathway regulated by RAB5 but do not include RAB7. The orange arrows indicate the AP-3-dependent pathway. The purple arrows indicate the ER to vacuole trafficking pathway. ER, endoplasmic reticulum; Golgi, Golgi apparatus; TGN, *trans*-Golgi network; MVB, multivesicular body; PM, plasma membrane; CW, cell wall. **(B)** Some special regulator proteins in bryophytes and species genome databases are used in this article. The complete table was in the Supplementary Tables. The numbers represent the quantity of the ortholog proteins in this species. The genome database reference for *Arabidopsis thaliana* (Lamesch et al., 2011), *P. patens* (Lang et al., 2018), *C. purpureus* (Carey et al., 2021), *M. polymorpha* (Bowman et al., 2017), *A. agrestis* (Li et al., 2020), *A. angustus* (Zhang et al., 2020), *A. punctatus* (Li et al., 2020). The *S. fallax* and *S. magellanicum* databases were obtained from DOE-JGI, http://phytozome.jgi.doe.gov/.

Extensive studies have demonstrated that, similar to in other eukaryotes, the conserved key molecular toolkit belonging to CCMM is in charge of vacuole biogenesis from multiple pathways in plants, mainly including ESCRT mediated MVB pathway (Gao et al., 2014; Kolb et al., 2015), Rab5/Rab7 mediated endosome maturation pathway (Ebine et al., 2014) and AP3-dependent pathway (Uemura and Ueda, 2014; Minamino and Ueda, 2019; Cui et al., 2020) (Figure 2A). Through the comparative genomic study of these key molecular toolkit genes in bryophytes, we found that, in line with this hypothesis, orthologs of most vacuole biogenesis genes are present in bryophytes. More notably, we found several significant gene loss events, which may be caused by reductive evolution for niche adaptation (Supplementary Tables 1, 2). We classified these gene loss events into four groups according to their molecular and cellular functions, namely the VPS9 protein, AP3 complex, KEG protein, and the MVB-related proteins (Figure 2B). In *A. thaliana*, most genes of these highlighted four groups are indispensable for seedling viability. It has been known that gene loss depends on the dispensability of gene function, which is affected by both the mutational robustness and environmental adaptability. Here we review what has been inferred about the molecular toolkit changes that accompanied the evolution of the vacuole biogenesis pathways in bryophytes, aiming to highlight the role that bryophytes could play in better understanding of vacuole biogenesis and function during land plant evolution.

The absence of VPS9 in all sequenced hornwort is of great interest because in *Arabidopsis, vps9* null mutants are defective in early embryo development with the fragmented vacuole, which highlights the necessity of its function (Goh et al., 2007). The function of VPS9 was first identified in yeast S. cerevisiae as Rab5 guanidine exchange factors (GEF) to generate an active Rab5-GTP complex (Prag et al., 2003). Rab protein activation by GTP binding via GEF protein is a prerequisite to complete vesicle targeting and fusion. For Rab5 activation, studies in yeast and human identified Vps9p and Rabex-5 as GEFs and found the shared region of homology now known as the Vps9 domain (Carney et al., 2006). From yeast to humans and plants, the small GTPase Rab5/Rab7 switch is crucial for the endosomal maturation pathway. During the maturation process, the early endosomal Rab5 GTPase is replaced with the late endosomal Rab7 GTPase (Figure 2B). *Arabidopsis* genome encodes three members of RAB5, and the activation of RAB5 via VPS9a enables subsequent cellular events (Kotzer et al., 2004). The action of the VPS9 protein enhances the RAB5-MON1-CCZ1 complex formation which will further run the pathway with the assistance of RAB7 (Minamino and Ueda, 2019; Cui et al., 2020; Hu et al., 2020). Despite the importance of VPS9a protein in many land plants, there has not been any VPS9a protein in three members of the *Anthoceros* family, including *Anthoceros agrestis, Anthoceros angustus*, and *Anthoceros punctants* (Figure 2B and Supplementary Table 1). This suggests the diverged evolution of VPS9 mediated vacuole biogenesis in hornworts.

Different from RAB5/RAB7 pathway, the adaptor protein complex 3 (AP-3) mediated an independent vacuole trafficking pathway in yeast, flies, mammals, and seed plants (Boehm and Bonifacino, 2002). The transportation of VAMP711 and VAMP713 in *A. thaliana* is carried out through the AP-3 pathway (Feng et al., 2017; Takemoto et al., 2018; Cui et al., 2020). The AP-3 complex comprises four members including, AP-3β, AP-3δ, AP-3μ, and AP-3σ. In particular, *M. polymorpha* lacks the AP-3δ protein, while *A. angustus* lack the AP-3σ. However, AP-3δ or AP-3μ in *A. agrestis* that grown in two different environments show an opposite manner which requires further investigation. Similarly, no VPS16 protein, was identified in *A. agrestis* which indicates the alteration or diversified manner of AP3 pathway in these species (Figure 2B and Supplementary Table 1).

ESCRT-regulated MVB formation is critical for vacuole biogenesis and function in both yeast and seed plants (Gao et al., 2017). Before being transported to vacuoles, protein cargoes are sequestered into ILVs with the help of ESCRT machinery. Plants contain ESCRT-I, -II, -III, and VPS4 (vacuole protein sorting 4) isoforms as the yeast and mammal, but no ESCRT-0 subunits VPS27 and Hse1 (Hurley and Hanson, 2010). Instead, there were TOL1-9 (TOM1-like) proteins in *A. thaliana* and bryophytes (Korbei et al., 2013), which function similarly to ESCRT-0. However, we were able to find ISTL1 (Goodman et al., 2021) in *A. thaliana* and yeast, but not in these bryophytes or mammal (Figure 2B and Supplementary Table 2). Specially, VPS37 (component of ESCRT-I) (Okumura et al., 2013), LIP5 (positive regulator of VPS4/SKD1) (Buono et al., 2016), and FYVE4 (affect the connection of ESCRT-III and VPS4) (Liu et al., 2021) are absent in hornwort. More interestingly, as a suppressor of FREE1 (Gao et al., 2014), RESURRECTION1 (RST1) (Zhao et al., 2019) only exists in *S. fallax, S. magellanicum* and *M. polymorpha*, which indicates that this protein may have special functions in these bryophytes (Figure 2B).

KEG is a plant-specific RING-type E3 ligase, that localizes on the early endosome, and plays an essential role in multiple endomembrane trafficking processes including vacuole biogenesis (Gu and Innes, 2012). Here we found the specific KEG gene loss in *P*. *patens* and *C. purpureus*, indicating that KEG mediated pathway can be bypassed in these species.

## Conclusions and Perspectives

This review summarized the current understanding of bryophyte vacuole morphology and function. Combining with our comparative phylogenetic analysis of vacuole biogenesis-related CCMM genes, so far, we are sure that bryophytes share key vacuole-related features with seed plants, but with several exceptional innovations.

Similarly like in seed plants, the different populations of vacuoles exist in bryophytes, however, there lack of clear evidence to support the extant of PSV in bryophytes. In addition, similar vacuole fission and fusion dynamics have been observed in both seed plants and bryophytes. The interesting point is the prominent effect of ABA on vacuole dynamics in bryophytes, while the underlying mechanism and the biological significance are still mysterious.

Studies in seed plants, especially in Arabidopsis, have reached several different models for vacuole biogenesis (Cui et al., 2020). Whether a similar different *de-novo* biogenesis model (Cui et al., 2019) and ER-derived model (Viotti et al., 2013) for vacuole formation co-exist in bryophyte still need further research.

Secondary metabolites, especially UVAC, may represent a unique innovation in bryophytes' vacuole function. Under the UV-B radiation, bryophyte vacuoles would accumulate SUVAC, which may protect the plant from UV-B damage (Fabón et al., 2012; Hespanhol et al., 2014; Monforte et al., 2015; Soriano et al., 2019).

Usually one would assume that the propensity for gene loss negatively correlated with the necessity of the function on organism viability. That is to say, if a gene is kept throughout the whole span of evolution, that is because the function of this gene is essential for survival. However, here we found that some essential proteins are absent in *Anthoceros*, such as VPS9a, VPS37, LIP5, FYVE4, and RST1, and some other proteins disappeared in *S. fallax, S. magellanicum, P. patens*, and *M. polymorpha* (Figure 2B). How is the vacuole trafficking and biogenesis running in these plants without these key proteins? Whether there are some alternative proteins to help sort cargoes? It remains to be answered. To answer these questions, we strongly urge that on the basis of further in-depth study of model bryophytes, we also need to pay attention to more evolutionary basal or advanced bryophytes.

## Author Contributions

H-rL, CS, DH, W-qF, Z-yW, YL, R-lZ, and QZ reviewed literature, formulated ideas, and wrote the manuscript. H-rL and QZ prepared the figures. H-rL, CS, and QZ conducted the bioinformatic analysis. All authors contributed to the article and approved the submitted version.

## Funding

Sponsored by East China Normal University and Shanghai Pujiang Program 20PJ1403200.

## Conflict of Interest

The authors declare that the research was conducted in the absence of any commercial or financial relationships that could be construed as a potential conflict of interest.

## Publisher's Note

All claims expressed in this article are solely those of the authors and do not necessarily represent those of their affiliated organizations, or those of the publisher, the editors and the reviewers. Any product that may be evaluated in this article, or claim that may be made by its manufacturer, is not guaranteed or endorsed by the publisher.
