# Supplementary material for: Vacuoles in Bryophytes: Properties, Biogenesis, and Evolution
Source: Front Plant Sci. 2022 Jun 7;13:863389. doi: 10.3389/fpls.2022.863389 (PMC9209779; doi:10.3389/fpls.2022.863389)
Supplement: Supplementary file 1 [file Data_Sheet_1.PDF]

Supplementary Tables

Supplementary Table 1. Proteins related to vacuole trafficking pathway in different species.

Regulators	Proteins	Yeast	Mammal	*Arabidopsis thaliana*	*Sphagnum fallax v1.1*	*Sphagnum magellanicum v1.1*	*Physcomitrella patens v3.3*	*Ceratodon purpureus GG1 v1.1*	*Ceratodon purpureus R40 v1.1*	*Marchantia polymorpha v3.1*	*Anthoceros agrestis* (BONN)	*Anthoceros agrestis* (OXF)	*Antheoceros angustus*	*Anthoceros punctatus*
PI3K complex	ATG6	1	1	1	1	1	2	0	1	1	1	1	1	1
	VPS34	1	1	1	1	1	1	1	1	1	1	1	1	1
	VPS15	1	1	1	2	2	2	1	1	1	1	1	1	1
	VPS38	1	1	1	1	1	1	1	1	1	1	1	1	1
RAB5 and its GEF	RAB5	3	4	3	3	3	5	1	1	4	3	3	3	3
	VPS9	2	6	2	2	2	1	1	1	1	0	0	0	0
RAB7 and its GEF	RAB7	1	1	8	3	3	4	1	1	1	1	1	1	1
	MON1	1	1	1	1	1	2	1	1	1	2	2	2	2
	CCZ1	1	1	2	1	1	1	1	1	1	1	1	1	1
HOPS	VPS11	1	1	1	1	1	1	1	1	1	1	1	1	1
	VCL/VPS16	1	1	1	1	1	1	1	1	1	0	0	1	1
	VPS18	1	1	1	1	1	1	2	2	1	1	1	1	1
	VPS33	1	1	1	1	1	1	1	1	1	1	1	1	1
	VPS39	1	1	1	1	1	1	1	1	1	1	1	1	1
	VPS41	1	1	1	2	2	1	1	1	1	1	1	1	1
SNARE complex	VTI11	1	1	1	3	3	2	2	2	1	2	2	2	2
	SYP22	1	0	1	3	6	2	2	2	1	1	1	1	1
	SYP51	1	0	1	1	1	1	1	1	1	1	1	1	1
	VAMP727	1	1	1	7	7	2	2	2	1	2	2	2	1
	VAMP711/713			2	2	2	3	2	2	1	1	1	1	1
AP-3	AP-3β	1	1	1	1	1	2	1	1	1	1	1	1	1
	AP-3 δ	1	1	1	1	1	1	1	1	0	1	0	1	1
	AP-3 μ	1	1	1	1	1	1	1	1	1	0	1	1	1
	AP-3 σ	1	1	1	1	1	1	1	1	1	2	2	0	1
others	KEG	0	0	1	1	1	0	0	0	1	1	1	1	1
