# Supplementary material for: Vacuoles in Bryophytes: Properties, Biogenesis, and Evolution
Source: Front Plant Sci. 2022 Jun 7;13:863389. doi: 10.3389/fpls.2022.863389 (PMC9209779; doi:10.3389/fpls.2022.863389)
Supplement: Supplementary file 2 [file Data_Sheet_2.PDF]

Supplementary Table 2. ESCRT components in different species.

Regulators	Proteins	Yeast	Mammal	*Arabidopsis thaliana*	*Sphagnum fallax v1.1*	*Sphagnum magellanicum v1.1*	*Physcomitrella patens v3.3*	*Ceratodon purpureus GG1 v1.1*	*Ceratodon purpureus R40 v1.1*	*Marchantia polymorpha v3.1*	*Anthoceros agrestis* (BONN)	*Anthoceros agrestis* (OXF)	*Antheoceros angustus*	*Anthoceros punctatus*
ESCRT-0	VPS27	1	1	0	0	0	0	0	0	0	0	0	0	0
	Hse1	1	1	0	0	0	0	0	0	0	0	0	0	0
	TOL1/2	0	4	2	3	3	2	1	1	1	0	1	1	1
	TOL3-9	0		7	4	6	2	2	1	1	1	1	0	1
ESCRT-I	VPS23	1	1	2	2	2	2	1	1	2	1	1	2	1
	VPS28	1	1	2	1	1	2	1	1	1	1	1	2	1
	VPS37	1	4	2	3	3	3	2	2	1	0	0	0	0
	MVB12	1	1	0	0	0	0	0	0	0	0	0	0	0
ESCRT-II	VPS22	1	1	1	1	1	1	1	1	1	1	1	1	1
	VPS25	1	1	1	1	1	1	1	1	1	1	1	1	1
	VPS36	1	1	1	1	1	1	1	1	1	1	1	1	1
ESCRT-III and accessory proteins	VPS20	1	1	2	1	1	1	1	1	1	2	2	1	1
	SNF7	1	3	2	5	5	4	2	2	2	2	3	2	3
	VPS24	1	1	2	2	2	2	2	3	1	1	1	1	1
	VPS2	1	2	3	5	6	4	3	2	3	2	2	2	2
	CHMP1	1	3	2	6	6	4	3	3	2	1	1	1	1
	VPS60	1	1	2	2	2	2	1	1	1	1	1	1	1
	ISTL1	1	0	1	0	0	0	0	0	0	0	0	0	0
	CHMP7	1	1	1	1	1	1	1	1	1	0	1	0	1
VPS4 and accessory proteins	VPS4	1	1	1	4	4	2	1	1	1	1	1	1	1
	LIP5	1	1	1	2	2	1	2	2	1	0	0	0	0
Others	FYVE4	0	0	1	1	1	1	1	1	1	0	0	0	0
	FREE1	0	0	1	3	2	1	1	1	1	1	1	1	1
	RST1	0	1	1	1	1	0	0	0	1	0	0	0	0
	AtBRO1	1	1	1	2	3	2	1	1	1	1	1	1	1
	Bro1L			2	3	3	6	4	3	0	1	1	1	1
	AMSH	1	1	3	2	2	3	2	2	1	1	1	1	0
